# Effects of adductor canal block versus femoral nerve block in patients with anterior cruciate ligament reconstruction: A protocol for a systematic review and meta-analysis

**DOI:** 10.1097/MD.0000000000016763

**Published:** 2019-09-06

**Authors:** Xuwen Liu, Jiawen Zhou, Guping Mao, Qiao Yu, Xin Wu, Hong Sun, Hua Yang

**Affiliations:** a Department of Clinical Medical College, Guizhou Medical University, Guiyang; b Department of Joint Surgery, First Affiliated Hospital of Sun Yat-sen University, Guangzhou; c Department of Orthopaedics, Guiqian International General Hospital; d Department of Orthopaedics, Affiliated Hospital of Guizhou Medical University, Guiyang, PR China.

**Keywords:** adductor canal block, anterior cruciate ligament reconstruction, femoral nerve block, pain relief, quadriceps strength

## Abstract

**Objective:**

It is reported that both adductor canal block (ACB) and femoral nerve block (FNB) are commonly used methods for postoperative analgesia in anterior cruciate ligament (ACL) reconstruction. Currently, no record has compared the efficacy of postoperative pain relief and the influence to quadriceps strength between them. This study aims to provide a protocol to compare the efficacy and safety between ACB and FNB for the postoperative analgesia of ACL reconstruction.

**Methods:**

This study will be performed in accordance with the guideline of the Preferred Reporting Items for Systematic Review and Meta-analysis Protocols. Online databases including PubMed, Embase, Web of Science, Cochrane Library, Wanfang database, and the Chinese National Knowledge Infrastructure database will be systematically searched from their inception up May 31, 2019. All randomized controlled trials will be included in present meta-analysis. The quality of enrolled literatures will be evaluated by using the Cochrane Collaboration Risk of bias Tool. Statistical analysis will be calculated by the Review Manager 5.3.

**Results:**

This review will investigate the efficacy and safety of ACB compared with FNB in patients undergoing ACL reconstruction. The primary outcomes are visual analog scale, cumulative opioid consumption during 24 hours after surgery, numerical rating scale, and the time to first straight-leg raise. The secondary outcomes include maximal voluntary isometric contraction, stretching torque at 3, 6 months’ follow-up, and adverse effects.

**Conclusion:**

Findings of this systematic review and meta-analysis will summarize the current evidence in postoperative analgesia for ACL reconstruction and also provide implications for clinical practice.

## 1 Introduction

Anterior cruciate ligament (ACL) reconstruction is a common procedure in sports medicine which is often accompanied by considerable early postoperative pain.^[1]^ Generally, patients with ACL reconstruction have expectations for a rapid return to daily life. The inadequate postoperative pain relief can lead to poor joint mobility and thus result in the development of adhesions, weakened ligament insertion, and muscle atrophy. Therefore, postoperative pain management plays a critical role in the rehabilitation of patients undergoing ACL reconstruction.^[2]^ Recently, peripheral nerve blocks such as femoral nerve block (FNB) and adductor canal block (ACB) for postoperative pain management in ACL reconstruction have become popular because of their ability to reduce requirements of opioids and the risk of opioid related side effects.^[3]^

FNB shows superior analgesia when compared with placebo, and continuous intra-articular and wound infusion after ACL reconstruction.^[4–6]^ Furthermore, subjects with FNB have a reduction of opioid requirements in knee surgery.^[7]^ Although FNB plays an important role in postoperative analgesia after ACL reconstruction, its influence to quadriceps strength remains controversial. Magnussen et al demonstrated that FNB leaded to quadriceps strength reduction and poorer KOOS symptom subscale score at 6 weeks following ACL reconstruction.^[8]^ In addition, patients treated with FNB after ACL reconstruction had significant isokinetic deficits in knee extension and flexion strength at 6 months.^[9]^ Conversely, Stebler et al reported that a continuous FCB was unable to result in worsened functional outcomes after ACL reconstruction.^[10]^ ACB is a motor-sparing method which mainly blocks the saphenous nerve and the nerve to vastus medialis while passing through adductor canal.^[11]^ It is well established that ACB is a valuable adjunct for post-operative analgesia after major knee surgery.^[12]^ Similar to local infiltration analgesia, ACB performs a satisfactory pain analgesia after ACL reconstruction.^[13,14]^

Numerous systematic review and meta-analyses have compared the efficacy of pain control and the influence to quadriceps strength between them in total arthroplasty.^[15–18]^ To the best of our knowledge, there is no systematic review and meta-analysis that compares the effect of analgesia and the influence to quadriceps strength between ACB and FCB after ACL reconstruction. Currently, several RCTs have investigated the discrepancy between them in pain relief and functional recovery after ACL reconstruction.^[19–23]^ However, consensus regarding the optimal management of postoperative pain and quadriceps strength in this setting is still lacking. The aim of this study is to systematically review available RCTs to assess the efficacy of postoperative analgesia and the influence to quadriceps strength of ACB compared to FCB after ACL reconstruction.

## 2 Methods

### 2.1 Study registration

This study protocol has been registered in the PROSPERO and the registration number is CRD42019134810. This study will be performed and reported following the Preferred Reporting Items for Systematic Reviews and Meta-Analyses diagnostic test accuracy criteria.

### 2.2 Ethics

Ethical approval or patient consent is not required because the present study is a review of previously published articles.

### 2.3 Eligibility criteria

The eligibility criteria are summarized using PICOS approach (patients, intervention, comparisons, outcome, and study design type).

#### 2.3.1 Participants

Patients undergoing primary or revision ACL reconstruction will be included. There will be no restrictions on age, gender, and ethnicity.

#### 2.3.2 Interventions

The patients in intervention group are treated with ACB after ACL reconstruction.

#### 2.3.3 Comparisons

The patients in control group receive FNB after ACL reconstruction.

#### 2.3.4 Outcomes

The primary outcomes include visual analog scale (VAS), cumulative opioid consumption during 24 hours after surgery, numerical rating scale (NRS), and the time to first straight-leg raise. The secondary outcomes are maximal voluntary isometric contraction, stretching torque at 3, 6 months’ follow-up, and adverse effects such as postoperative infection, vomiting, and arthrofibrosis.

#### 2.3.5 Study design

RCTs published with no language restriction up to May 31, 2019 will be considered eligible for our study.

### 2.4 Data sources and search strategy

We will follow the Preferred Reporting Items for Systematic Review and Meta-analysis Protocols (PRISMA) statement to report this system review. PubMed (1966–May 2019), Embase (1980–May 2019), and Cochrane Library (1966–May 2019) databases is comprehensively searched. The key words include “anterior cruciate ligament reconstruction,” “femoral nerve block,” or “adductor block,” and “randomized controlled trials (RCTs).” The following articles including case reports, reviews, retrospective studies, letters, and animal experimental studies will be excluded in present study.

### 2.5 Study selection and data extraction

#### 2.5.1 Study selection

**Figure 1:**
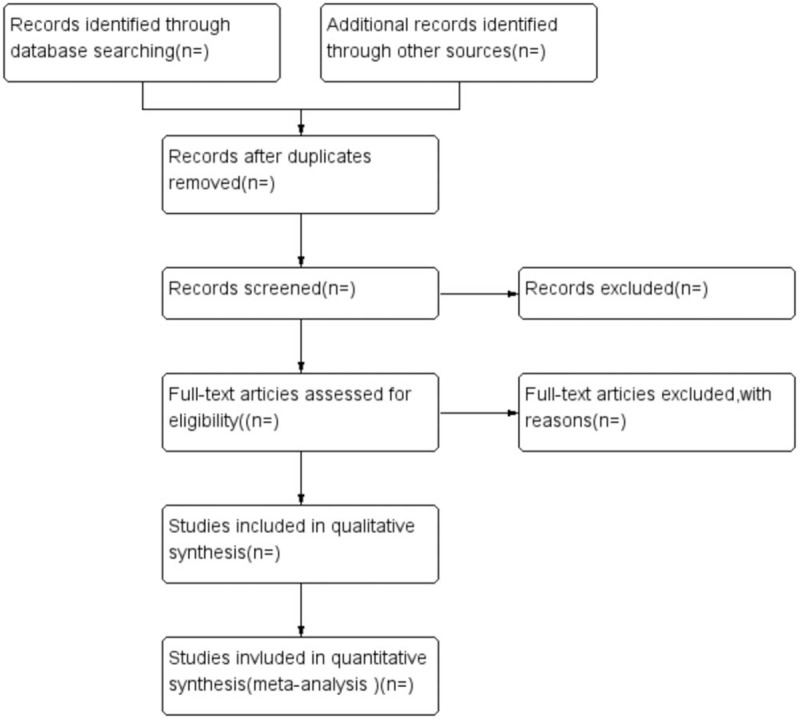
The flowchart of study selection.

Initial search records will be performed by 2 reviewers (XL and JZ) independently. Two reviewers will independently screen the titles and abstracts. The full text will be read if records meet the predefined inclusion criteria. The flowchart of study selection is shown in Figure 1.

#### 2.5.2 Data extraction

Two investigators (XL and JZ) will independently extract the following data from the included literatures: surname of the first author, publication year, sample size, sex ratio, age, and detail methods in each group. All study characteristics will be summarized in the same standardized collection form. Any disagreement between investigators will be resolved by discussion. When necessary, a third investigator (HS) will help reach a consensus with all investigators.

### 2.6 Risk of bias assessment

Two authors will evaluate the risk of bias of each RCT by the Cochrane Risk of Bias tool.^[24]^ Each article will be assessed based on the following 7 items: allocation concealment, double blindness, incomplete outcome, selective reporting, randomization process, and measurements of outcomes and other bias. Each item will be described as a low risk of bias, a high risk of bias, or an unclear risk of bias.

### 2.7 Statistical analysis

All statistical analyses will be conducted by Review Manager 5.3(Cochrane Collaboration, Software Update, Oxford, UK). Continuous data will be assessed using mean difference (MD) with corresponding 95% confidence interval (CIs). Dichotomous data will be calculated using relative risk and 95% CIs. *P* value <.05 is regarded as statistically significant. The statistics and quantity of heterogeneity will be estimated depending on the value of *P* (*P*_*Q*_) and *I*^2^ using the standard *χ*^2^ test and *I*^2^ statistic, respectively. When *I*^2^ >50% and *P*_*Q*_ < .1, the heterogeneity will be considered to be significant and then a random effect-model will be used. Otherwise, a fixed-effect model will be chosen.

## 3 Discussion

ACL reconstruction is often associated with moderate-to-severe postoperative pain. A favorable analgesia will improve the satisfaction of patients undergoing ACL reconstruction. ACB and FNB are 2 commonly used methods for postoperative analgesia after ACL reconstruction. Recently, there are several prospective clinical trials exploring the analgesia and strength in patients with ACB or FNB, whereas the results are still controversial. It is necessary to perform such systematic review and meta-analysis to analysis the difference between ACB and FNB. To our knowledge, this is the first systematic review and meta-analysis to compare ACB with FNB for the efficacy of pain management and the influence to quadriceps strength in patients with ACL reconstruction. This study will be conducted in accordance with the guideline of the PRISMA. The result will bring a comprehensive comparison to determine which option is better for the postoperative analgesia after ACL reconstruction. The findings of this study may provide helpful evidence for clinical practice.

## Acknowledgments

The authors thank Prof. Xianwen Shang and Hao Zhao at Affiliated Hospital of Guizhou Medical University (Guiyang Guizhou, China) for the assistance of writing and language editing.

## Author contributions

**Conceptualization:** Guping Mao, Hua Yang.

**Data curation:** Jiawen Zhou.

**Formal analysis:** Xuwen Liu, Jiawen Zhou, Guping Mao, Qiao Yu.

**Funding acquisition:** Xin Wu.

**Methodology:** Xuwen Liu, Hong Sun, Hua Yang.

**Project administration:** Hong Sun.

**Software:** Xuwen Liu, Jiawen Zhou.

**Supervision:** Qiao Yu, Xin Wu, Hua Yang.

**Writing – original draft:** Xuwen Liu, Hong Sun, Hua Yang.

**Writing – review & editing:** Hong Sun, Hua Yang.

Hua Yang orcid: 0000-0002-2133-3408.

## References

[1] Beck PR Nho SJ Balin J Postoperative pain management after anterior cruciate ligament reconstruction. J Knee Surg 2004;17:18–23. 14971669 10.1055/s-0030-1247142

[2] Secrist ES Freedman KB Ciccotti MG Pain management after outpatient anterior cruciate ligament reconstruction: a systematic review of randomized controlled trials. Am J Sports Med 2016;44:2435–47. 26684664 10.1177/0363546515617737

[3] Xu J Chen XM Ma CK Peripheral nerve blocks for postoperative pain after major knee surgery. Cochrane Database Syst Rev 2014;12:Cd010937. 10.1002/14651858.CD010937.pub2 25501884

[4] Dauri M Fabbi E Mariani P Continuous femoral nerve block provides superior analgesia compared with continuous intra-articular and wound infusion after anterior cruciate ligament reconstruction. Reg Anesth Pain Med 2009;34:95–9. 19282706 10.1097/AAP.0b013e31819baf98

[5] Wulf H Lowe J Gnutzmann KH Femoral nerve block with ropivacaine or bupivacaine in day case anterior crucial ligament reconstruction. Acta Anaesthesiol Scand 2010;54:414–20. 20085546 10.1111/j.1399-6576.2009.02200.x

[6] Dauri M Polzoni M Fabbi E Comparison of epidural, continuous femoral block and intraarticular analgesia after anterior cruciate ligament reconstruction. Acta Anaesthesiol Scand 2003;47:20–5. 12492792 10.1034/j.1399-6576.2003.470104.x

[7] Fenten MGE Bakker SMK Scheffer GJ Femoral nerve catheter vs local infiltration for analgesia in fast track total knee arthroplasty: Short-term and long-term outcomes. Br J Anaesth 2018;121:850–8. 30236246 10.1016/j.bja.2018.05.069

[8] Magnussen RA Pottkotter K Stasi SD Femoral nerve block after anterior cruciate ligament reconstruction. J Knee Surg 2017;30:323–8. 27362929 10.1055/s-0036-1584538

[9] Luo TD Ashraf A Dahm DL Femoral nerve block is associated with persistent strength deficits at 6 months after anterior cruciate ligament reconstruction in pediatric and adolescent patients. Am J Sports Med 2015;43:331–6. 25466410 10.1177/0363546514559823

[10] Stebler K Martin R Kirkham KR Electrophysiological study of femoral nerve function after a continuous femoral nerve block for anterior cruciate ligament reconstruction: A randomized, controlled single-blind trial. Am J Sports Med 2017;45:578–83. 27836905 10.1177/0363546516669715

[11] Vora MU Nicholas TA Kassel CA Adductor canal block for knee surgical procedures: review article. J Clin Anesth 2016;35:295–303. 27871547 10.1016/j.jclinane.2016.08.021

[12] Lund J Jenstrup MT Jaeger P Continuous adductor-canal-blockade for adjuvant post-operative analgesia after major knee surgery: preliminary results. Acta Anaesthesiol Scand 2011;55:14–9. 21039357 10.1111/j.1399-6576.2010.02333.x

[13] Thapa D Ahuja V Pandey K Evaluation of analgesic efficacy of dexmedetomidine as adjuvant with ropivacaine in ultrasound-guided adductor canal block in patients following anterior cruciate ligament reconstruction surgeries. Br J Pain 2019;13:91–8. 31019690 10.1177/2049463718796865 PMC6463348

[14] Stebler K Martin R Kirkham KR Adductor canal block versus local infiltration analgesia for postoperative pain after anterior cruciate ligament reconstruction: a single centre randomised controlled triple-blinded trial. Br J Anaesth 2019;123:e343–9. 31130273 10.1016/j.bja.2019.04.053 PMC6676236

[15] Kuang MJ Ma JX Fu L Is adductor canal block better than femoral nerve block in primary total knee arthroplasty? A grade analysis of the evidence through a systematic review and meta-analysis. J Arthroplasty 2017;32:3238–48. 28606458 10.1016/j.arth.2017.05.015

[16] Gao F Ma J Sun W Adductor canal block versus femoral nerve block for analgesia after total knee arthroplasty: a systematic review and meta-analysis. Clin J Pain 2017;33:356–68. 27322397 10.1097/AJP.0000000000000402

[17] Wang D Yang Y Li Q Adductor canal block versus femoral nerve block for total knee arthroplasty: a meta-analysis of randomized controlled trials. Sci Rep 2017;7:40721. 28079176 10.1038/srep40721 PMC5228345

[18] Dong CC Dong SL He FC Comparison of adductor canal block and femoral nerve block for postoperative pain in total knee arthroplasty: a systematic review and meta-analysis. Medicine (Baltimore) 2016;95:e2983. 27015172 10.1097/MD.0000000000002983 PMC4998367

[19] Abdallah FW Whelan DB Chan VW Adductor canal block provides noninferior analgesia and superior quadriceps strength compared with femoral nerve block in anterior cruciate ligament reconstruction. Anesthesiology 2016;24:1053–64. 10.1097/ALN.0000000000001045 26938989

[20] Lynch JR Okoroha KR Lizzio V Adductor canal block versus femoral nerve block for pain control after anterior cruciate ligament reconstruction: a prospective randomized trial. Am J Sports Med 2019;47:355–63. 30557034 10.1177/0363546518815874

[21] El Ahl MS Femoral nerve block versus adductor canal block for postoperative pain control after anterior cruciate ligament reconstruction: a randomized controlled double blind study. Saudi J Anaesth 2015;9:279–82. 26240546 10.4103/1658-354X.154708 PMC4478820

[22] Runner RP Boden SA Godfrey WS Quadriceps strength deficits after a femoral nerve block versus adductor canal block for anterior cruciate ligament reconstruction: a prospective, single-blinded, randomized trial. Orthop J Sports Med 2018;6: 2325967118797990. 10.1177/2325967118797990 PMC6158619 30276220

[23] Ghodki PS Shalu PS Sardesai SP Ultrasound-guided adductor canal block versus femoral nerve block for arthroscopic anterior cruciate ligament repair under general anesthesia. J Anaesthesiol Clin Pharmacol 2018;34:242–6. 30104837 10.4103/joacp.JOACP_172_17 PMC6066890

[24] Higgins JP Altman DG Gotzsche PC The Cochrane Collaboration's tool for assessing risk of bias in randomised trials. BMJ 2011;343:d5928. 22008217 10.1136/bmj.d5928 PMC3196245

